# Live Imaging of Nitric Oxide Dynamics Reveals Cell Type-Specific NO Signaling in Air–Liquid Interface Cultures of Human Sinonasal Epithelial Cells

**DOI:** 10.3390/biomedicines14061340

**Published:** 2026-06-12

**Authors:** Sakura Hirokane, Keiichiro Kiyohara, Sachio Takeno, Tsuyoshi Sugimoto, Tomohiro Kawasumi, Yukako Okamoto, Rikuto Fujita, Chie Ishikawa, Yuichiro Horibe, Takashi Ishino, Takao Hamamoto, Tsutomu Ueda

**Affiliations:** Department of Otorhinolaryngology, Head and Neck Surgery, Graduate School of Biomedical Sciences, Hiroshima University, Hiroshima 734-8551, Japan; sakura16@hiroshima-u.ac.jp (S.H.); kkiyo@hiroshima-u.ac.jp (K.K.); b221385@hiroshima-u.ac.jp (T.S.); kwtm2022@hiroshima-u.ac.jp (T.K.); kakomama@hiroshima-u.ac.jp (Y.O.); rikto@hiroshima-u.ac.jp (R.F.); chie0324@hiroshima-u.ac.jp (C.I.); horibey@hiroshima-u.ac.jp (Y.H.); tishino@hiroshima-u.ac.jp (T.I.); takao0320@hiroshima-u.ac.jp (T.H.); uedatsu@hiroshima-u.ac.jp (T.U.)

**Keywords:** paranasal sinus, chronic rhinosinusitis, nasal polyps, ciliated cells, nitric oxide, nitric oxide synthase, air–liquid interface culture, mucociliary clearance

## Abstract

**Background/Objectives**: Chronic rhinosinusitis with nasal polyps (CRSwNP) is associated with epithelial remodeling, impaired mucociliary clearance, and altered nitric oxide (NO) metabolism. However, cell type-specific mechanisms underlying epithelial NO signaling remain poorly understood. This study investigated NO-related signaling in differentiated human sinonasal epithelial cells. **Methods**: Human sinonasal tissues were obtained from patients with CRSwNP (*n* = 20) and control subjects (*n* = 20). Air–liquid interface (ALI) cultures were established from donor-derived epithelial cells. Ciliated and non-ciliated cells were identified by immunostaining for acetylated α-tubulin and BCAM. Expression of inducible nitric oxide synthase (iNOS) and endothelial nitric oxide synthase (eNOS) was analyzed by quantitative RT-PCR. Intracellular NO-related fluorescence signals were evaluated using DAF-FM fluorescence imaging. **Results**: CRSwNP tissues exhibited significantly increased iNOS expression and elevated iNOS/eNOS ratios, whereas eNOS expression did not differ significantly from that in controls. ALI cultures reproduced differentiated sinonasal epithelium containing both ciliated and non-ciliated cell populations. DAF-FM fluorescence signals were significantly higher in ciliated cells than in non-ciliated cells (80.3 ± 25.3 vs. 49.3 ± 21.1). Non-selective NOS inhibition markedly reduced fluorescence signals in both cell types, whereas selective iNOS inhibition reduced but did not abolish signals in ciliated cells. **Conclusions**: NO-related signaling appears to differ among epithelial cell subtypes. Persistence of fluorescence signals after selective iNOS inhibition suggests a contribution of constitutive NOS activity in ciliated cells, whereas non-ciliated cells appear to rely more heavily on iNOS-dependent pathways. These findings support the hypothesis that altered epithelial NO signaling contributes to epithelial dysfunction and impaired mucociliary homeostasis in CRSwNP.

## 1. Introduction

Chronic rhinosinusitis (CRS) is a chronic inflammatory disease of the nasal cavity and paranasal sinuses characterized by persistent symptoms such as nasal obstruction, rhinorrhea, and olfactory dysfunction. CRS is increasingly recognized not as a single disease entity, but as a heterogeneous group of disorders with distinct inflammatory profiles and molecular mechanisms [1,2,3,4,5]. In particular, the importance of endotype-based classification centered on type 2 inflammation has been emphasized in chronic rhinosinusitis with nasal polyps (CRSwNP) [6,7,8].

Impaired mucociliary clearance is considered a key pathological feature of CRS. Airway cilia play an essential role in host defense by removing inhaled pathogens and foreign particles through coordinated mucociliary transport. However, persistent inflammation and infection in CRS disrupt ciliary function, leading to impaired mucociliary clearance and perpetuation of chronic inflammation. Recent advances in single-cell RNA sequencing analyses have revealed a reduction in ciliated epithelial cells, epithelial differentiation abnormalities, and metabolic dysregulation in CRSwNP, highlighting the importance of epithelial remodeling in disease pathogenesis [9,10,11,12,13,14].

Nitric oxide (NO) is an important signaling molecule in the airway epithelium that exerts antimicrobial activity and enhances ciliary motility, both of which are essential for maintaining mucociliary clearance [15,16,17,18]. NO is synthesized primarily by nitric oxide synthase (NOS), and two NOS isoforms, endothelial NOS (eNOS) and inducible NOS (iNOS), play major roles in the airway epithelium [19]. Under physiological conditions, low concentrations of NO derived from eNOS contribute to the regulation of ciliary motility. In contrast, sustained high-output NO production by iNOS may contribute to oxidative stress and tissue injury under inflammatory conditions. Furthermore, eNOS has been shown to be specifically expressed in ciliated epithelial cells of the respiratory tract and to regulate ciliary beating through the NO–cGMP signaling pathway [20]. These findings suggest that epithelial-derived NO plays a central role in maintaining mucociliary transport.

Interestingly, although decreased nasal nitric oxide (nNO) levels have been consistently reported in patients with CRSwNP, increased iNOS expression has also been observed at the tissue level [21,22]. These findings suggest that NO abnormalities in CRS may not simply reflect reduced NO production, but rather qualitative alterations in NO signaling associated with changes in the balance of NOS isoforms. However, whether NO production and NOS isoform activity differ between ciliated and non-ciliated epithelial cells remains unclear.

Therefore, we established an air–liquid interface (ALI) culture model of human sinonasal epithelial cells to investigate cell type-specific differences in NO production and the contribution of NOS isoforms to NO signaling. We further evaluated the effects of selective NOS inhibition to better understand the mechanisms underlying epithelial NO regulation and their potential relevance to CRSwNP pathophysiology.

## 2. Materials and Methods

### 2.1. Study Design and Tissue Samples

All procedures in this study complied with the ethical standards outlined in the Helsinki Declaration. The study protocol was approved by the Institutional Review Board of Hiroshima University School of Medicine (approval no. E2014-9136; approval date: 2 November 2022). Written informed consent was obtained from all participants prior to inclusion.

We enrolled patients with chronic rhinosinusitis with nasal polyps (CRSwNP) and control subjects who underwent endoscopic endonasal sinus surgery between April 2024 and August 2025. Tissue samples were collected from the ethmoid sinus and nasal polyps during surgery. The diagnosis of CRS was based on patients’ history, clinical symptoms, nasal endoscopic findings, and computed tomography (CT) images [1]. Patients who had previously undergone sinus surgery were excluded. None of the participants had received systemic or topical corticosteroids for 4 weeks prior to surgery. None of the patients received antibiotic therapy before surgery. Antihistamines and leukotriene receptor antagonists were continued only in patients who required these medications for concomitant diseases such as allergic rhinitis (AR) or bronchial asthma (BA). None of the patients routinely performed saline nasal irrigation before surgery. In addition, no patients received biologic therapy during the study period. Allergic rhinitis was diagnosed according to clinical history, nasal symptoms, positive nasal eosinophil findings, and positive allergen-specific IgE antibodies against common airborne allergens. Regarding clinical phenotypes, no patients were diagnosed with NSAID-exacerbated respiratory disease (N-ERD), allergic fungal rhinosinusitis (AFRS), or cystic fibrosis. Control subjects were patients without sinus infection who underwent endonasal surgery and had normal paranasal sinus mucosa with normal radiological findings.

The study consisted of two sequential experimental phases. First, tissue-based analyses, including quantitative RT-PCR and immunohistochemistry, were performed using specimens obtained from both control subjects and patients with CRSwNP to characterize NOS isoform expression in vivo. Second, functional analyses of NO-related signaling were conducted using air–liquid interface (ALI) cultures established exclusively from CRSwNP-derived sinonasal epithelial cells, allowing investigation of cell type-specific NO-related fluorescence signals under controlled in vitro conditions.

### 2.2. Air-Liquid Interface (ALI) Culture

Human sinonasal epithelial cells (HSNECs) were isolated from surgical specimens obtained from patients with CRSwNP and cultured under air–liquid interface (ALI) conditions. Donors used for ALI culture experiments represented a subset of the CRSwNP cohort included in the tissue-based analysis. They were selected based on the availability of sufficient viable tissue for primary cell isolation and successful differentiation under ALI conditions. Passage-2 HSNECs were seeded onto Transwell inserts and differentiated for up to 4 weeks following establishment of the air–liquid interface. Morphological, molecular, and functional analyses were performed during this differentiation period, and NO imaging studies were conducted after 4 weeks of ALI culture.

HSNECs were isolated by enzymatic dissociation in minimal essential medium (MEM; MilliporeSigma, St. Louis, MO, USA) containing 1.0 mg/mL protease (MilliporeSigma) and 0.01 mg/mL DNase (MilliporeSigma) for 24 h at 4 °C, followed by washing with bronchial epithelial growth medium (BEGM; Lonza, CC-3170, Walkersville, MD, USA). The epithelial cells were collected by centrifugation using a cell scraper and seeded on tissue culture dishes coated with type I collagen (IWAKI, Shizuoka, Japan) in BEGM supplemented with 100 U/mL penicillin and 100 μg/mL streptomycin. The cultures were maintained at 37 °C in a humidified atmosphere with 5% CO_2_. The culture medium was replaced every 2–3 days. Primary sinonasal epithelial cells were cultured for 6–7 days until reaching 80% confluence (1.7–4.0 × 10^6^ cells/dish).

Passage-2 HSNECs (1.5 × 10^5^ cells/well) were seeded for ALI culture in 0.5 mL of medium on Transwell clear culture inserts (12 mm diameter, 0.4 μm pore size; Costar, Corning Inc., Corning, NY, USA), as previously described [23]. Initially, cells were cultured under submerged conditions in BEGM with supplements for 5–7 days. After confluence was achieved, the apical medium was removed to establish the air–liquid interface. Thereafter, cells were maintained in Air–Liquid Interface Epithelial Differentiation Medium (Cat. No. LHB-LM0050; Lifeline Cell Technology, Frederick, MD, USA), a fully supplemented medium specifically optimized for mucociliary differentiation of human airway epithelial cells. The medium contains L-glutamine, recombinant human epidermal growth factor (rhEGF), hydrocortisone hemisuccinate, recombinant human insulin, epinephrine, transferrin, triiodothyronine, and all-trans retinoic acid, which support epithelial maturation and ciliogenesis under ALI conditions. Basal medium was replenished every 2–3 days throughout the differentiation period.

### 2.3. Scanning Electron Microscopy (SEM)

ALI culture specimens were fixed in 2.5% glutaraldehyde and 2% paraformaldehyde in 0.1 M phosphate buffer (pH 7.4). After fixation, samples were rinsed three times in distilled water, post-fixed in 2% osmium tetroxide for 2 h, and dehydrated through a graded ethanol series (50%, 70%, 80%, 90%, 95%, and 100%). Specimens were then immersed in tert-butanol for 1.5 h and dried using a t-butanol freeze-drying device (VFD-21S; Vacuum Device Inc., Ibaraki, Japan). The dried samples were sputter-coated with osmium (Neoc-ST; Meiwa Forsys, Tokyo, Japan) and examined using a JSM-7800F scanning electron microscope (JEOL Ltd., Tokyo, Japan). Cell types were identified, and their relative proportions were calculated as mean percent surface area. Ciliary synchronization, defined as the percentage of cilia “in phase” with the metachronal pattern, was evaluated after whole-surface scanning as described elsewhere [24].

### 2.4. Quantitative RT-PCR Analysis

Samples were preserved in RNAlater™ (Thermo Fisher Scientific, Waltham, MA, USA). Quantitative RT-PCR was performed using an ABI Prism 7300 system (Applied Biosystems, Foster City, CA, USA) as previously described [25]. Total RNA was extracted using RNeasy Mini Kits (Qiagen, Valencia, CA, USA) and reverse-transcribed to cDNA using a High-Capacity RNA-to-cDNA Kit (Applied Biosystems).

Gene expression was quantified using TaqMan Gene Expression Assays (Thermo Fisher Scientific) for iNOS (NOS2) (Hs01075529_m1), eNOS (NOS3) (Hs01574665_m1), and BCAM (Hs00170663_m1). GAPDH (Hs02786624_g1) served as an internal control. Relative expression was calculated using the comparative Ct (2^−ΔΔCt^) method and expressed as the ratio of target gene to GAPDH expression.

### 2.5. Immunohistology and Immunofluorescence Microscopy

Primary antibodies included anti-human iNOS (NOS2) mouse monoclonal antibody (clone 2D2-B2; R&D Systems, Minneapolis, MN, USA), anti-human eNOS (NOS3) rabbit polyclonal antibody (Thermo Scientific, Fremont, CA, USA), anti-human Arl13B rabbit polyclonal antibody (#17711-1-AP; Proteintech, Rosemont, IL, USA; 1:500), anti-BCAM (basal cell adhesion molecule) mouse monoclonal antibody (#sc-365191; Santa Cruz Biotechnology, Dallas, TX, USA; 1:100) and anti-human acetylated α-tubulin mouse monoclonal antibody (#SC-23950; Santa Cruz Biotechnology, Dallas, TX, USA; 1:500).

Immunostaining for NOS isoforms was performed on 5-μm-thick cryostat sections. For antigen retrieval, sections were immersed in Histo VT One (Nacalai Tesque, Kyoto, Japan) at 70 °C for 40 min. The sections were then incubated overnight at 4 °C in the presence of the primary antibodies. The color development was performed using the streptavidin-biotin amplification technique. Peroxidase activity was visualized by the diaminobenzidine solution. Sections were counterstained with Mayer’s hematoxylin. Negative controls were performed omitting the primary antibody or using an isotype control antibody from the same species.

Samples from ALI cultures were fixed in 4% paraformaldehyde for 20 min at room temperature. Non-specific binding was blocked by incubation with 10% goat serum for 1 h. Cultures were incubated overnight at 4 °C with the primary antibodies, followed by the appropriate secondary antibodies—anti-mouse goat IgG Alexa Fluor^®^ 555 (#A32727; Invitrogen, Carlsbad, CA, USA; 1:100) and anti-rabbit IgG goat Alexa Fluor^®^ 488 (#A32731; Invitrogen; 1:100). Nuclei were counterstained with DAPI (#71-03-00; KPL, Gaithersburg, MD, USA) in PBST containing 0.01% sodium azide for 1 h. After washing with PBS, samples were mounted in VECTASHIELD Antifade Mounting Medium (#H-1000-10; Vector Laboratories, Newark, CA, USA) and imaged using a laser scanning confocal microscope (Stellaris 5; Leica, Wetzlar, Germany). IgG1 isotype controls confirmed the absence of non-specific staining.

### 2.6. Live Cell Imaging of Nitric Oxide (NO)-Related Fluorescence Signals

Live-cell imaging of NO-related fluorescence signals was performed as described previously [26,27,28,29]. ALI cultures were incubated on the apical side with 10 μM DAF-FM diacetate (#SK1004-01; Goryo Chemical, Sapporo, Japan) in HBSS for 30 min. DAF-FM has optimal excitation and emission wavelengths of 495 and 515 nm, respectively. The incubation time and concentration of DAF-FM were determined based on preliminary experiments and published data [30]. To stimulate NO production, L-arginine was added to the solution at a final concentration of 0.5 mM.

Differential interference contrast (DIC) microscopy was used simultaneously to identify ciliated cell-rich regions in ALI cultures. To confirm NO production by specific cell types, double-label immunofluorescence for acetylated tubulin was performed on DAF-FM-loaded samples fixed in 4% paraformaldehyde in 0.1 M phosphate buffer (PB) for 30 min at 20 °C. To assess the contribution of each NOS isoform to NO production, ALI cells were treated during DAF-FM incubation with either 0.5 mM NG-nitro-L-arginine methyl ester (L-NAME), a non-specific NOS inhibitor, or 100 μM 1400W, a selective iNOS inhibitor.

To visualize intracellular NO-related signals, fluorescence imaging using the NO-sensitive fluorescent probe DAF-FM was performed, and fluorescence intensity was quantitatively analyzed to compare NO production capacity between epithelial cell subtypes [31]. Using this approach combined with immunohistochemistry and laser scanning confocal microscopy (LSCM), we identified heterogeneity in NO-related fluorescence and bioavailability among epithelial cell subtypes, including basal and ciliated cells, providing new insights into the spatial regulation of NO signaling within the sinonasal epithelium.

### 2.7. Quantitative Image Analysis

Fluorescence intensities of DAPI, DAF-FM, and acetylated tubulin were quantified using ImageJ software version 1.54 (NIH, Bethesda, MD, USA) as described previously [32]. For quantitative analysis, regions of interest (ROIs) corresponding to individual epithelial cells were defined using the ROI Manager and standardized to 1924 pixels per ROI. Mean fluorescence intensity was measured for each ROI. Cells positive for acetylated α-tubulin were classified as ciliated cells. Cells lacking detectable acetylated α-tubulin staining were categorized as non-ciliated epithelial cells. This population may include basal, secretory, goblet, and intermediate epithelial cell types. For donor-level analysis, fluorescence measurements obtained from multiple ROIs were averaged within each donor. Comparisons between ciliated and non-ciliated cells were subsequently performed using donor-level mean fluorescence values.

### 2.8. Statistical Analysis

Statistical analyses were performed using GraphPad Prism software version 8.0 (GraphPad Software, San Diego, CA, USA). Data distribution was assessed using the Shapiro–Wilk test. For comparisons between two independent groups, the Mann–Whitney U test was used. For comparisons among more than two groups, the Kruskal–Wallis test followed by Dunn’s multiple-comparison test was used. For time-dependent comparisons during ALI culture, repeated-measures one-way ANOVA with Geisser–Greenhouse correction was used, followed by multiple-comparison testing where appropriate. Comparisons between ciliated and non-ciliated cells were performed using donor-level mean values and analyzed using a paired *t*-test. The statistical test used for each analysis is indicated in the corresponding figure legend. A *p* value < 0.05 was considered statistically significant.

## 3. Results

### 3.1. Comparison of NOS Isoform mRNA and Protein Expression in CRSwNP Patients

The demographic and clinical characteristics of the study population are summarized in Table 1. No significant differences were observed between the control group (*n* = 20) and the CRSwNP group (*n* = 20) with respect to age, sex, body mass index (BMI), prevalence of allergic rhinitis and bronchial asthma, current smoking status, or peripheral blood eosinophil counts. In contrast, tissue eosinophil counts were significantly higher in the CRSwNP group than in the control group (*p* < 0.001). In addition, CT scores were significantly elevated in the CRSwNP group, reflecting increased disease severity (Table 1).

**Figure 1:**
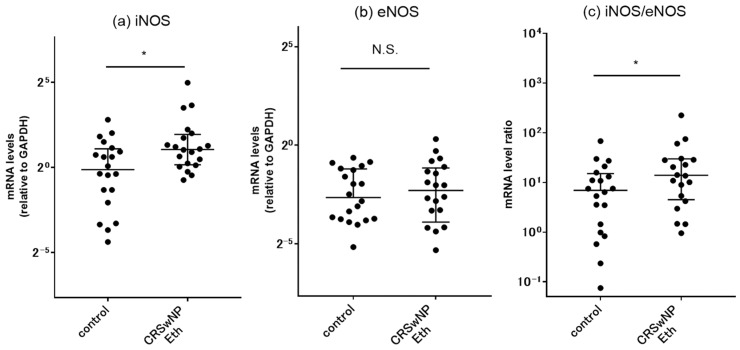
Comparison of NOS isoform mRNA expression in ethmoid sinus mucosa from control subjects (*n* = 20) and patients with CRSwNP (*n* = 20) as determined by quantitative RT-PCR. (**a**) iNOS (NOS2) mRNA expression, (**b**) eNOS (NOS3) mRNA expression, and (**c**) the iNOS/eNOS expression ratio. Expression levels were normalized to GAPDH. Center lines indicate median values and error bars represent interquartile ranges. Statistical comparisons were performed using the Mann–Whitney U test. iNOS expression was significantly increased in the CRSwNP group (*p* = 0.0195), whereas eNOS expression did not differ significantly between groups (*p* = 0.8831). The iNOS/eNOS ratio was significantly increased in the CRSwNP group (*p* = 0.0491). CRS, chronic rhinosinusitis; Eth, ethmoid sinus; NP, nasal polyps. * *p* < 0.05; N.S., not significant.

mRNA expression levels of NOS isoforms (iNOS and eNOS) in ethmoid sinus mucosa were analyzed by RT-PCR (Figure 1). iNOS expression was significantly increased in the CRSwNP group compared with the control group, whereas no significant difference in eNOS expression was observed between the two groups. Furthermore, the iNOS/eNOS ratio was significantly elevated in the CRSwNP group.

**Figure 2:**
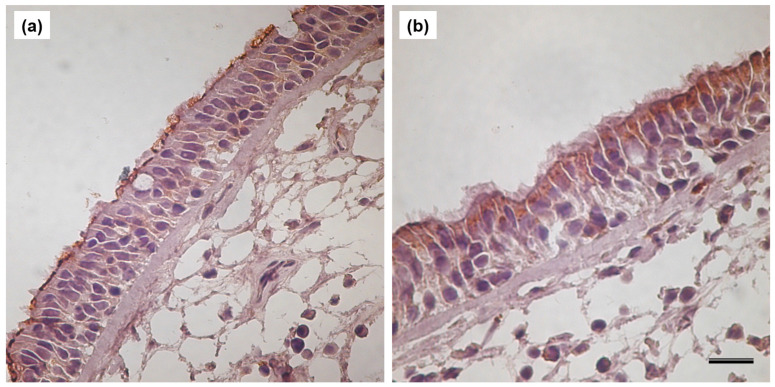
Representative immunohistochemical images showing the expression of (**a**) iNOS and (**b**) eNOS in nasal polyp tissue obtained from a patient with CRSwNP. Scale bars: 20 μm.

Figure 2 shows representative immunohistochemical images illustrating the distribution of iNOS- and eNOS-positive cells in nasal polyp mucosa. Positive iNOS immunoreactivity was predominantly observed in epithelial cells, mainly within the cytoplasm. In addition, numerous inflammatory cells distributed throughout the submucosal region, particularly eosinophils in the ethmoid sinus mucosa, also exhibited positive staining. In contrast, eNOS immunoreactivity was consistently detected in ciliated epithelial cells as well as in endothelial cells lining capillary vessels.

### 3.2. Phenotypic Identification and Morphological Evaluation of ALI-Cultured Cells

All ALI cultures analyzed in this study were established using sinonasal epithelial cells derived from patients with CRSwNP. To investigate the differentiation process toward ciliated epithelial cells and their NO-producing capacity in vitro, primary sinonasal epithelial cells were isolated from surgical specimens obtained during sinus surgery and expanded in basal cell growth medium prior to differentiation under air–liquid interface (ALI) conditions. After enzymatic dissociation, cells were seeded at high density onto permeable Transwell filters, and the apical medium was removed on the following day. This procedure exposed the luminal surface of epithelial cells to air and induced differentiation from basal cells into ciliated epithelial cells.

**Figure 3:**
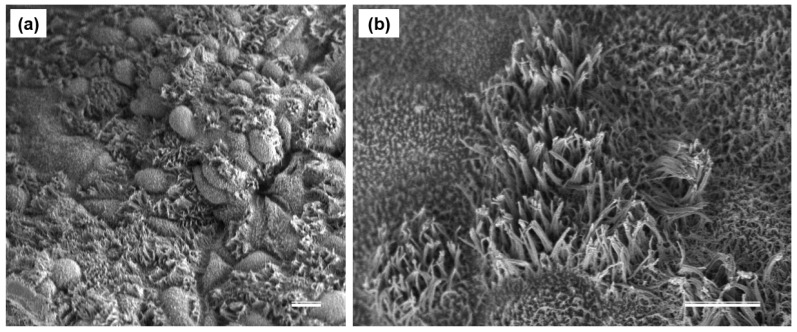
Representative SEM photomicrographs showing surface morphology and cell types of ALI cultured cells. (**a**) The SEM image at lower magnification at week 4 of culture shows approximately half of the surface epithelium is covered with ciliated cells. (**b**) The SEM image at higher magnification shows a cluster of ciliated cells. In these cells, ciliary orientation appeared heterogeneous and less uniformly aligned than typically observed in native sinonasal epithelium. Scale bars: 10 μm.

To determine whether the sinonasal epithelial phenotype was preserved during ALI culture, surface morphology and protein/gene expression profiles were evaluated. Scanning electron microscopy (SEM) demonstrated epithelial cells arranged in a cobblestone-like pattern with characteristic microvilli typical of respiratory epithelium (Figure 3). Low-magnification images obtained after 4 weeks of ALI culture showed that approximately half of the epithelial surface was covered by ciliated cells. High-magnification images revealed clustered ciliated cells. Ciliary orientation appeared heterogeneous and less uniformly aligned compared with that typically observed in native sinonasal epithelium.

The proportion of ciliated cells reached a maximum at 3–4 weeks after ALI induction. These findings were consistent with a previous report demonstrating that human sinonasal epithelial cells differentiate into respiratory epithelium under ALI conditions and require approximately 14 days for mature ciliogenesis [33].

**Figure 4:**
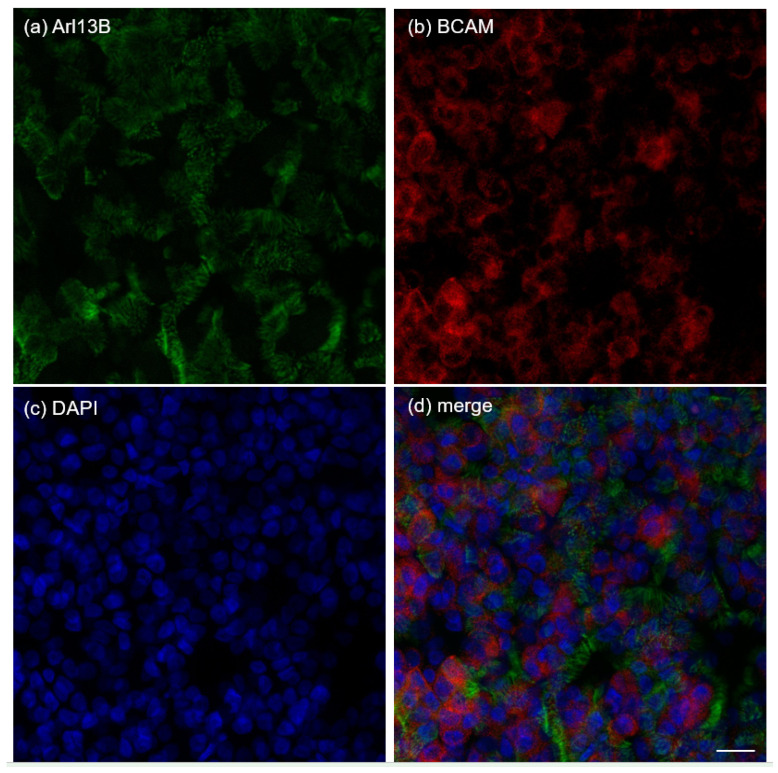
Phenotypic characterization of human sinonasal epithelial cells in ALI culture using cell-specific immunofluorescent markers. (**a**–**d**) Representative immunofluorescence images of human sinonasal epithelial cells in ALI culture for 4 weeks. Cells show robust expression of Arl13B (green), a marker of primary cilia, and BCAM (red), a marker of basal cells, merged with DAPI (nuclei, blue), confirming the coexistence of Arl13B-positive and BCAM-positive epithelial cell populations in ALI cultures. Scale bars: 15 μm.

To confirm the presence of specific epithelial subtypes, immunofluorescence staining was performed using Arl13B as a marker for primary cilia and BCAM as a marker for basal cells. Positive staining for both markers confirmed the presence of ciliated and basal cell populations within the ALI cultures (Figure 4). After 4 weeks of culture, tightly packed epithelial cells with a cobblestone-like arrangement and apical microvillar structures were observed by laser scanning confocal microscopy. Nuclear staining with DAPI identified cell nuclei (blue), while abundant Ac-α-tubulin-positive ciliated cells labeled with Alexa Fluor Plus 555 (red) were also detected. These findings were reproducibly observed in all ALI culture preparations.

### 3.3. Time-Dependent Changes in Gene Expression During ALI Culture

**Figure 5:**
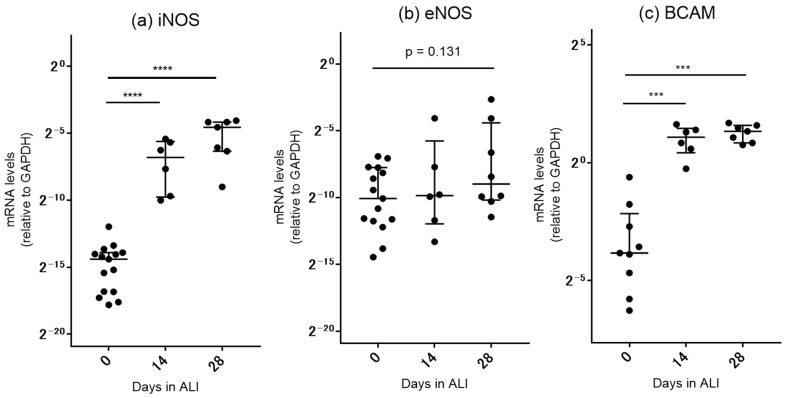
Gene expression of primary sinonasal epithelial cells in ALI cultures. (**a**) iNOS, (**b**) eNOS, and (**c**) BCAM mRNA expression during 4 weeks of differentiation as determined by qPCR. Data points represent the average values obtained from five independent donor-derived ALI cultures. Data are presented as mean ± SEM. Statistical comparisons among time points were performed using repeated-measures one-way ANOVA with Geisser–Greenhouse correction, followed by multiple-comparison testing. Significant increases in iNOS and BCAM expression were observed during differentiation, whereas eNOS expression showed no significant change (*p* = 0.131). *** *p* < 0.001; **** *p* < 0.0001.

Temporal changes in gene expression during ALI culture were analyzed by quantitative PCR (qPCR) (Figure 5). Using GAPDH as an internal control, mRNA expression levels of iNOS and BCAM increased significantly over time. In contrast, although eNOS expression showed an increasing trend, the change did not reach statistical significance (*p* = 0.131). These results demonstrated a time-dependent increase in iNOS and BCAM mRNA expression during ALI culture, whereas eNOS expression showed a non-significant increasing trend.

### 3.4. Comparison of NO-Related Fluorescence Signals Between Epithelial Cell Subtypes

**Figure 6:**
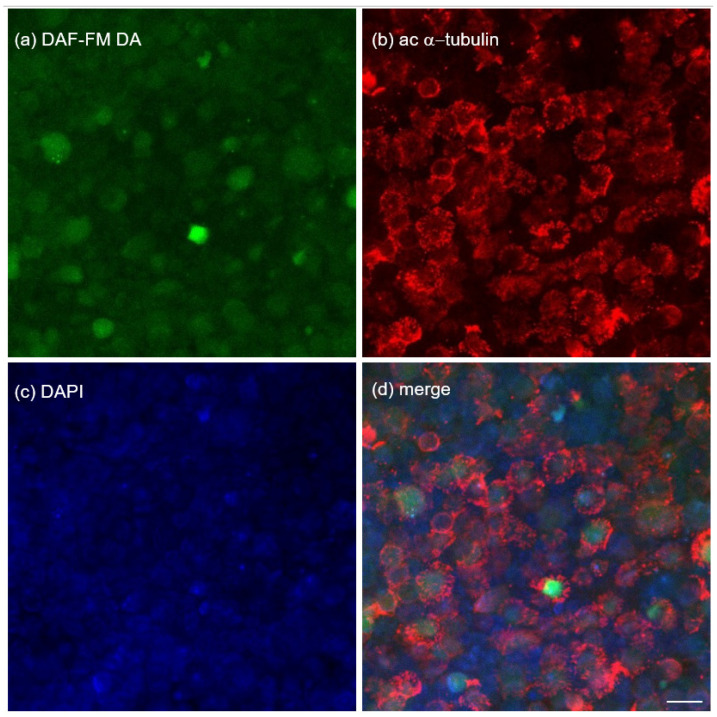
Nitric oxide production in human sinonasal epithelial cells in ALI culture using NO-specific fluorescent indicators DAF-FM DA. Representative LSCM fluorescence images of ALI cultures after 4 weeks of differentiation. (**a**) Cells show distinct NO-related fluorescence as detected by DAF-FM fluorophores (green). (**b**) AlexaFluor 555-labeled staining of ac-tubulin (red) shows abundant apical cilia with a cobblestone epithelial pattern. (**c**) Nuclear DAPI stain is shown in blue. Scale bars: 15 μm.

Intracellular NO production in ALI-cultured cells after 4 weeks of differentiation was evaluated using the NO-sensitive fluorescent probe DAF-FM DA (Figure 6). Addition of the NOS substrate L-arginine (0.5 mM) enhanced intracellular green fluorescence intensity, suggesting increased intracellular NO-related signaling. Furthermore, double fluorescence staining with Ac-α-tubulin demonstrated spatial overlap between DAF-FM fluorescence signals and ciliated epithelial cells.

**Figure 7:**
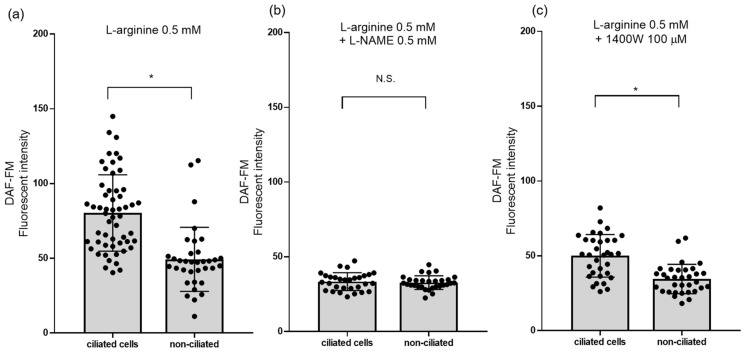
Comparison of nitric oxide (NO)-related fluorescence signals between ciliated and non-ciliated sinonasal epithelial cells in 4-week ALI cultures as revealed by DAF-FM fluorescence. Each data point represents the mean fluorescence intensity measured within a single region of interest (ROI; 1924 pixels) corresponding to an individual cell. ROIs were obtained from ALI cultures derived from independent donors. Bar graphs show mean ± SEM. (**a**) Administration of 0.5 mM L-arginine increased DAF-FM fluorescence intensity, with ciliated cells exhibiting significantly higher fluorescence than non-ciliated cells. (**b**) The non-selective NOS inhibitor L-NAME (0.5 mM) markedly reduced fluorescence signals in both cell types. (**c**) The selective iNOS inhibitor 1400W (100 μM) reduced fluorescence intensity but preserved significantly higher fluorescence in ciliated cells. Statistical comparisons between ciliated and non-ciliated cells were performed using donor-level mean fluorescence values (*n* = 5 donors) and analyzed using a paired *t*-test. * *p* < 0.05; N.S., not significant.

In the present study, ciliated and non-ciliated epithelial cells differentiated under ALI conditions were identified and compared with respect to NO production capacity. To evaluate the functional contribution of NOS isoforms in each cell type, a non-selective NOS inhibitor (L-NAME) and an iNOS-selective inhibitor (1400W) were used. 1400W is a highly selective inhibitor of iNOS. As shown in Figure 7, marked NO-related fluorescence was observed in ALI-cultured epithelial cells under L-arginine stimulation. When fluorescence measurements were averaged at the donor level, ciliated cells exhibited significantly higher DAF-FM fluorescence intensity than non-ciliated cells (paired *t*-test, *p* = 0.0018). DAF-FM fluorescence intensity was significantly higher in acetylated α-tubulin-positive ciliated cells (80.3 ± 25.3, mean ± SEM) than in non-ciliated cells (49.3 ± 21.1). In contrast, treatment with the non-selective NOS inhibitor L-NAME (0.5 mM for 1 h) markedly suppressed NO production, reducing fluorescence intensity to 33.4 ± 5.8 in ciliated cells and 32.8 ± 4.5 in non-ciliated cells, thereby abolishing the difference between the two cell types. Furthermore, although treatment with the iNOS-selective inhibitor 1400W (100 μM) reduced NO production, fluorescence intensity remained significantly higher in ciliated cells (50.0 ± 14.0) than in non-ciliated cells (34.5 ± 9.6).

## 4. Discussion

The present study demonstrated that nitric oxide (NO)-related signaling differs according to epithelial cell subtype in human sinonasal epithelium [30,34]. Three principal findings emerged from this study. First, CRSwNP tissues exhibited increased iNOS expression and an elevated iNOS/eNOS ratio compared with control mucosa. Second, differentiated air–liquid interface (ALI) cultures reproduced distinct ciliated and non-ciliated epithelial cell populations and showed time-dependent increases in NOS-related gene expression. Third, live-cell imaging using DAF-FM revealed significantly higher NO-related fluorescence signals in ciliated epithelial cells than in non-ciliated cells. Pharmacological inhibition studies further suggested that NO-related signals in ciliated cells involve contributions from both constitutive and inducible NOS pathways, whereas signals in non-ciliated cells were predominantly dependent on iNOS activity. These findings provide new evidence that NO-related signaling is spatially heterogeneous within the sinonasal epithelium and suggest that alterations in epithelial cell composition may influence the balance of NOS isoform activity in CRSwNP.

NO is synthesized from L-arginine by nitric oxide synthases (NOS), and three isoforms are present in mammals: nNOS (NOS1), iNOS (NOS2), and eNOS (NOS3) [35,36]. Although previous studies have reported little or no detectable nNOS expression in human sinonasal epithelial tissues, the present study focused on eNOS and iNOS, which are considered the principal NOS isoforms involved in NO production in the human sinonasal mucosa [16,37,38]. Under physiological conditions, eNOS primarily generates low concentrations of NO that regulate ciliary motility through the NO–cGMP signaling pathway [20]. In contrast, iNOS is induced by inflammatory stimuli and continuously produces high concentrations of NO, thereby contributing to inflammatory responses. In the present study, increased iNOS expression and an elevated iNOS/eNOS ratio were observed in the CRSwNP group, suggesting alterations in NOS isoform expression patterns under inflammatory conditions. Thus, the observed changes in NOS isoform expression may indicate alterations in the regulation of NO production in CRSwNP; however, direct functional changes in NO signaling were not assessed in the present study. Although physiological concentrations of NO enhance ciliary beat frequency (CBF), excessive NO production under inflammatory conditions may instead induce oxidative stress and secondary tissue injury, ultimately leading to ciliary dysfunction [15,39].

Air–liquid interface (ALI) culture is a widely used in vitro technique for establishing stable airway epithelial models that reproduce pseudostratified columnar epithelium containing ciliated cells. This system enables temporal analysis of differentiation from basal cells into ciliated cells, ciliogenesis, and associated changes in functional molecule expression while preserving three-dimensional epithelial architecture. Furthermore, ALI culture reproduces physiological properties of airway epithelium, including mucus secretion and mucociliary transport, making it a valuable model for investigating CRS pathophysiology [40]. In the present study, iNOS and BCAM expression increased significantly during ALI differentiation, whereas eNOS expression showed a non-significant increasing trend. These findings indicate progressive epithelial differentiation during ALI culture while suggesting differential regulation of NOS isoforms during this process.

Using DAF-FM fluorescence imaging, we observed significantly higher NO-related fluorescence signals in ciliated epithelial cells than in non-ciliated cells, suggesting that ciliated epithelial cells may contribute substantially to epithelial NO production. Because DAF-FM fluorescence reflects NO-related signals rather than direct quantification of NO production, these findings suggest heterogeneity in NO-related signaling among epithelial cell subtypes. Such cell type-dependent differences may be relevant to the maintenance of mucociliary function and epithelial homeostasis. DAF-FM DA is an activatable fluorescent probe that emits fluorescence upon reacting with NO. Because it enables conversion of short-lived NO into a stable fluorescent signal, DAF-FM has been widely used as an indicator of intracellular NO-related signals [28,29,31]. Although it should be noted that DAF-FM fluorescence represents a semi-quantitative NO-related signal and does not provide a direct or completely specific measurement of NO production.

In vivo fluorescence imaging using DAF-2DA has successfully visualized and quantitatively analyzed NO production in rat mesenteric microcirculation [41]. Moreover, analyses using L-NAME and the NOS1 inhibitor 7-NI demonstrated that NOS1-derived NO predominantly contributes to arteriolar function, whereas NOS3 (eNOS)-derived NO plays a major role in venular function, indicating that the physiological effects of NO are determined by the localization and functional differences among NOS isoforms. The pharmacological characteristics of 1400W, a highly selective iNOS inhibitor, have also been extensively investigated [42]. Overall, 1400W exerts potent and time-dependent inhibition against iNOS and exhibits tight binding with slow dissociation, whereas its inhibitory effects on eNOS and nNOS are relatively weak and reversible. Previous studies using murine ischemia–reperfusion models further demonstrated that selective iNOS inhibition by 1400W attenuates tissue injury in the renal cortex, whereas transient endothelial dysregulation may occur in the renal medulla, suggesting that the effects of iNOS inhibition depend on tissue-specific NO signaling balance and endothelial function [43,44].

In the present study, treatment with the non-selective NOS inhibitor L-NAME substantially reduced NO-related fluorescence signals in ciliated cells, whereas the iNOS-selective inhibitor 1400W did not completely suppress NO production. These findings suggest that NO production in ciliated cells involves multiple NOS isoforms, including both iNOS and eNOS. Importantly, the persistence of NO-related signals even under iNOS inhibition strongly suggests that constitutive NOS, mainly eNOS, contributes to basal NO production. Regulation of ciliary motility by NO has been reported to occur through the eNOS–NO–sGC–cGMP–PKG pathway [45]. The increase in CBF induced by L-arginine is suppressed by NOS inhibitors, soluble guanylate cyclase (sGC) inhibitors, and PKG inhibitors, supporting the importance of this signaling pathway. The cell type-dependent differences in NOS isoform expression and NO production observed in this study may reflect distinct functional roles in NO signaling. Specifically, physiological NO generated predominantly by eNOS in ciliated cells likely enhances ciliary motility through the cGMP–PKG pathway, whereas inflammatory NO production in non-ciliated cells is primarily iNOS-dependent and contributes less to ciliary motility regulation.

Furthermore, NO-mediated regulation of ciliary motility has been reported to depend on intracellular localization of signaling molecules [46]. Molecules including eNOS, guanylate cyclase (GC), PKG, and PKA localize near the axoneme and basal body. In particular, eNOS, PKG-I, and PKA-RII accumulate around basal bodies, whereas PKA-RI and PKG-II are distributed along the axoneme. Such localization suggests that NO production, cGMP generation, and PKG/PKA activation may function as an integrated localized signaling unit (“metabolon”) near the ciliary apparatus in the vicinity of cilia. Although the present findings are consistent with this concept, our study did not directly evaluate the subcellular localization of NOS isoforms or downstream signaling molecules. Therefore, the proposed spatial NO signaling model should be regarded as a hypothetical framework requiring further investigation.

In contrast, NO-related fluorescence in non-ciliated cells was almost completely abolished by 1400W, indicating that NO production in these cells is predominantly iNOS-dependent [42]. Because iNOS is induced by inflammatory cytokines and mediates sustained high-output NO production, these findings are consistent with NO production characteristics under inflammatory conditions.

The cell type-dependent differences in NO-related fluorescence observed in the present study are likely closely associated with spatially regulated NO signaling. In ciliated cells, eNOS and iNOS may cooperatively establish a localized NO signaling environment regulating ciliary motility, whereas non-ciliated cells lack such spatial regulation and predominantly generate inflammatory iNOS-derived NO. From this perspective, the reduction in ciliated cells in CRSwNP may represent not merely decreased NO production, but rather a disruption of the spatial regulation of NO signaling. Loss of the local physiological NO signaling environment may impair ciliary motility regulation, resulting in decreased mucociliary clearance and persistence of chronic inflammation. In addition, previous studies have demonstrated reduced nNO levels together with increased iNOS expression and increased proportions of non-ciliated cells in CRSwNP [47,48,49]. Taken together, these findings suggest that NO signaling may shift from promoting ciliary motility toward amplifying inflammation due to reduced eNOS-derived NO associated with ciliated cell loss and relative increases in inflammation-induced iNOS-derived NO.

Such qualitative alterations in NO signaling are likely involved in impaired mucociliary clearance and persistence of chronic inflammation [50]. These findings support the concept that qualitative dysregulation of NO signaling, rather than simple reductions in NO levels, contributes to epithelial dysfunction and chronic inflammation in CRSwNP. Collectively, ciliary dysfunction and epithelial remodeling in CRSwNP may involve two distinct abnormalities: (1) reduced physiological ciliary regulation mediated by eNOS-dependent NO, and (2) enhanced inflammation and tissue injury mediated by iNOS-dependent NO. These findings may provide a rationale for future studies investigating whether selective modulation of iNOS and eNOS activity could represent a potential therapeutic approach for CRSwNP.

Several limitations of this study should be acknowledged. First, this study was based on in vitro analyses using an air–liquid interface (ALI) culture system and therefore does not fully reproduce the in vivo environment. Accordingly, careful interpretation is required regarding the reproducibility of these findings in vivo. Second, it remains unclear whether increased iNOS-derived NO is a primary cause of inflammation or a secondary consequence of inflammatory processes. Additional studies incorporating temporal analyses and in vivo models will be necessary to clarify this issue. Third, the relatively small number of ALI culture preparations may limit the generalizability of the findings. Future studies with larger cohorts are warranted to validate the present results. Fourth, the non-ciliated cell population was defined by the absence of acetylated α-tubulin staining and therefore likely represents a heterogeneous mixture of basal, goblet, secretory, and intermediate epithelial cells. Further studies using additional lineage-specific markers will be necessary to define cell type-specific NO signaling more precisely. Fifth, concomitant type 2 inflammatory diseases such as allergic rhinitis and bronchial asthma, as well as the use of antihistamines and leukotriene receptor antagonists, may have influenced epithelial inflammatory status and NOS expression. Because the prevalence of these comorbidities was not completely matched between groups, residual confounding cannot be excluded.

In addition, concomitant allergic rhinitis, bronchial asthma, smoking status, and associated medical treatments may potentially influence epithelial inflammatory responses and NOS expression. Although the prevalence of these factors was generally comparable between groups and major confounding conditions such as N-ERD, AFRS, biologic therapy, and active infection were excluded, residual confounding cannot be completely ruled out. Another limitation is that the evaluation of ciliary synchronization and metachronal organization was based on static SEM images. Because SEM cannot directly assess dynamic ciliary motion, coordinated beating, or metachronal wave propagation, these observations should be interpreted as morphological rather than functional findings. Future studies using live-cell imaging and high-speed video microscopy will be required to characterize ciliary coordination more accurately.

## 5. Conclusions

The present study suggests that NO-related signaling in differentiated sinonasal epithelial cells may vary according to epithelial cell subtype. In ALI cultures, ciliated cells exhibited higher DAF-FM fluorescence signals than non-ciliated cells, and inhibitor experiments suggested differential contributions of NOS isoforms to these signals. Persistence of NO-related fluorescence after selective iNOS inhibition in ciliated cells may indicate a contribution from constitutive NOS activity, whereas the marked reduction observed in non-ciliated cells suggests greater dependence on iNOS-related pathways. These findings support the hypothesis that altered cell type-specific NO signaling may contribute to epithelial dysfunction and impaired mucociliary homeostasis in CRSwNP. Further studies incorporating protein-level validation, localization analyses of NOS isoforms, direct assessment of ciliary function, and in vivo confirmation are required to clarify the mechanisms underlying these observations.

## Figures and Tables

**Table 1:** Background and baseline characteristics of the study population.

Variable	Control (*n* = 20)	CRSwNP (*n* = 20)	*p* Value
Age (years)	47.4 ± 13.6	54.0 ± 11.1	0.101
Sex (male/female)	10/10	10/10	1.000
BMI (kg/m^2^)	23.9 ± 4.4	22.1 ± 2.5	0.127
Allergic rhinitis, *n* (%)	11 (55%)	14 (70%)	0.514
Bronchial asthma, *n* (%)	1 (5%)	4 (20%)	0.342
Current smoker, *n* (%)	3 (15%)	5 (25%)	0.695
CT score	NA	12.5 ± 4.8	—
Blood eosinophils (%)	2.6 (0.6–8.8)	4.45 (0.7–13.9)	0.129
Tissue eosinophils (cells/HPF)	3 (0–25)	74.2 (0.0–333.3)	<0.001

Data are presented as mean ± SD, median (range), or number (%). *p* values were calculated using Welch’s *t*-test, Mann–Whitney U test, or Fisher’s exact test, as appropriate according to data distribution.

## Data Availability

The data presented in this study are available on reasonable request from the corresponding author.

## Abbreviations

The following abbreviations are used in this manuscript:

ALI	air–liquid interface
AR	allergic rhinitis
BA	bronchial asthma
BCAM	basal cell adhesion molecule
CBF	ciliary beat frequency
CRS	chronic rhinosinusitis
CRSsNP	chronic rhinosinusitis without nasal polyps
CRSwNP	chronic rhinosinusitis with nasal polyps
DAF-FM DA	4-amino-5-methylamino-2′,7′-difluorofluorescein diacetate
GC	guanylate cyclase
HSNECs	human sinonasal epithelial cells
LSCM	laser scanning confocal microscopy
nNO	nasal nitric oxide
NO	nitric oxide
NOS	nitric oxide synthase
PCP	planar cell polarity
ROI	region of interest
SEM	scanning electron microscopy
